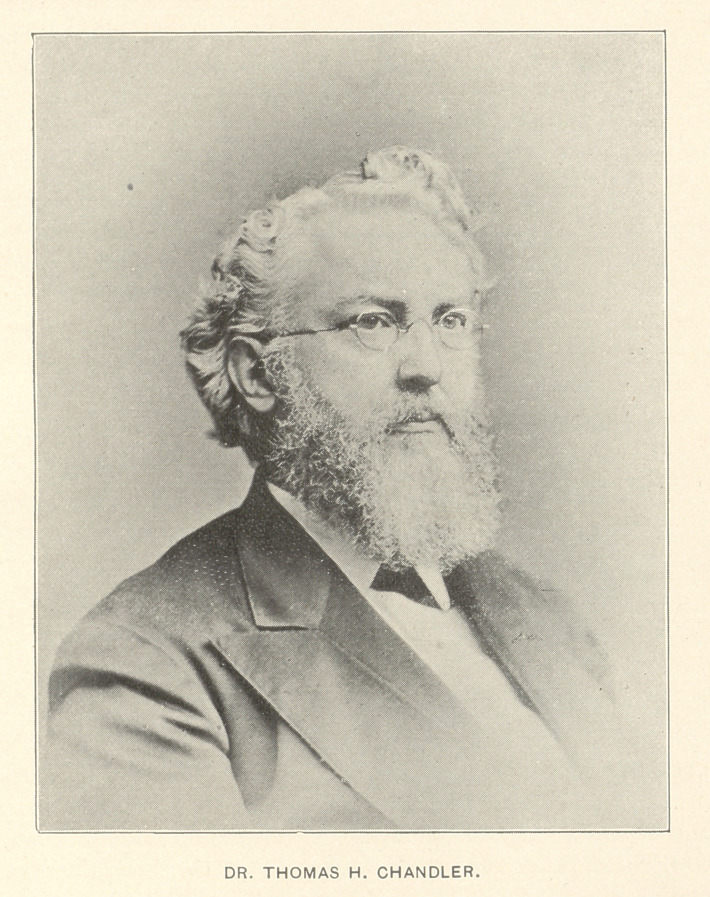# Thomas H. Chandler—Resolutions of Respect

**Published:** 1896-02

**Authors:** 


### Obituary.

### THOMAS H. CHANDLER—RESOLUTIONS OF RESPECT.

THE Boston Society for Dental Improvement have adopted the following resolutions of respect in behalf of the late Thomas H. Chandler, A.M., LL.B., M.D., D.M.D.:

*Resolved*, That in the death of Dr. Thomas H. Chandler, for twenty-one years the honored dean of the Harvard Dental School, the dental profession has lost one of its brightest ornaments and humanity one of its truest friends.

*Resolved*, That the Boston Society for Dental Improvement, recognizing our great loss, desire to place upon record our high appreciation of him as a man and a professional brother. He was a man of noble mien, with a character a fit complement of his person. His spotless character contained no trace of sordid ambition or self-laudation. Genuine and true, he had no sympathy with shams or any of the petty artifices of life. Gifted by nature, he adorned

DR. THOMAS H. CHANDLER.

those gifts with a most liberal education, and under the impulse of his love for sound learning he explored many fields of knowledge beyond the reach of a less cultivated mind. Painstaking and accurate in his investigations and conclusions, thorough and progressive in his profession, he became a safe guide to all who sought his counsel. Kind, generous, and sympathetic, he easily won the highest esteem of all lovers of true nobility. His very presence was a benediction; therefore be it

*Resolved*, That we will cherish his memory with the profoundest respect, and that under the inspiration of his noble example will strive to uplift the profession which he did so much to enrich.

*Resolved*, That we extend to his family our most heart-felt sympathy in this their great affliction, and would beg to remind them that, "although our heavenly Father's ways are often passed finding out," yet we know that "He doeth all things well."

*Resolved*, That a copy of these resolutions be forwarded to his family, and that they be placed upon our records.

BOSTON, December 3, 1895.

## Figures and Tables

**Figure f1:**